# On the transition from reconsolidation to extinction of contextual fear memories

**DOI:** 10.1101/lm.045724.117

**Published:** 2017-09

**Authors:** Lindsey F. Cassini, Charlotte R. Flavell, Olavo B. Amaral, Jonathan L.C. Lee

**Affiliations:** 1 School of Psychology, University of Birmingham, Birmingham, B15 2TT, United Kingdom; 2 Institute of Medical Biochemistry Leopoldo de Meis, Federal University of Rio de Janeiro, Rio de Janeiro, 21941-902, Brazil

## Abstract

Retrieval of an associative memory can lead to different phenomena. Brief reexposure sessions tend to trigger reconsolidation, whereas more extended ones trigger extinction. In appetitive and fear cued Pavlovian memories, an intermediate “null point” period has been observed where neither process seems to be engaged. Here we investigated whether this phenomenon extends to contextual fear memory. Adult rats were subjected to a contextual fear conditioning paradigm, reexposed to the context 2 d later for 3, 5, 10, 20, or 30 min, with immediate injections of MK-801 or saline following reexposure, and tested on the following day. We observed a significant effect of MK-801 with the 3- and 30-min sessions, impairing reconsolidation and extinction, respectively. However, it did not have significant effects with 5-, 10-, or 20-min sessions, even though freezing decreased from reexposure to test. Further analyses indicated that this is not likely to be due to a variable transition point at the population level. In conclusion, the results show that in contextual fear memories there is a genuine “null point” between the parameters that induce reconsolidation and extinction, as defined by the effects of MK-801, although NMDA receptor-independent decreases in freezing can still occur in these conditions.

The retrieval of an associative memory can result in different outcomes. Retrieval in the absence of further reinforcement can destabilize a memory, requiring a process of reconsolidation (Nader and Hardt 2009), or can cause memory extinction through new inhibitory learning (Giustino and Maren 2015). The balance between destabilization and extinction appears to be influenced by the relative strength of learning and extent of nonreinforced retrieval (Eisenberg et al. 2003; Suzuki et al. 2004; Lee et al. 2006; de la Fuente et al. 2011; Flavell and Lee 2013). More extensive stimulus reexposure (i.e., extinction training), or weaker initial conditioning is more likely to result in extinction, whereas more restricted stimulus reexposure preferentially engages memory destabilization. This apparent competition between destabilization and extinction manifests as a bidirectional effect of amnestic treatment, depending of the parameters of conditioning and retrieval. Either reconsolidation is impaired to reduce subsequent memory expression, or extinction is disrupted to maintain expression of the original memory (Eisenberg et al. 2003; Suzuki et al. 2004; Lee et al. 2006; de la Fuente et al. 2011; Flavell and Lee 2013).

In both appetitive Pavlovian and conditioned fear memories, recent evidence has indicated that extinction per se does not prevent memory destabilization and reconsolidation. In cue–sucrose, cue–fear, and context–fear settings, there appears to be a reexposure period between the parameters that engage destabilization and extinction, in which there is no behavioral effect of amnestic treatment (Flavell and Lee 2013; Merlo et al. 2014; Alfei et al. 2015). This “limbo” or “null point” suggests that extinction itself is not a boundary condition on reconsolidation. However, the interpretation of the negative effect at the null point is not straightforward. While it has been argued that only a three-phase transition model with a null point can explain the behavioral data (Merlo et al. 2014), this assumes that the parameters of transition are the same across individuals, thereby implying that the absence of a drug effect reflects a genuine null point at an individual level. However, it is also possible that, while at the individual level there is a gradual or step function transition from destabilization to extinction, there are individual differences in the parameters of that transition, resulting in a lack of group effect at intermediate points. Namely, at these intermediate reexposure conditions, some individuals could be undergoing a destabilization/reconsolidation process, while others would have transitioned into extinction learning. This might be expected to manifest as a greater variability in behavior due to the existence of different subgroups at the null point; however, this is unlikely to be identified with the sample sizes that have been previously used. In the current study, we used larger cohorts of rats and used multiple analytical approaches in order to confirm the existence of the null point effect for contextual fear memories (Alfei et al. 2015) and disambiguate its potential explanations, namely: (1) that the null point is a genuine effect and represents a phenomenon at the individual level or (2) that the null point is an artifact of variation in the transition point between reconsolidation and extinction at the population level.

## Results

### CFC memory is insensitive to MK-801 between the parameters that induce reconsolidation and extinction

In order to confirm the existence of the null point in the reactivation of contextual fear memories, Lister-Hooded rats were subjected to a contextual fear conditioning (CFC) paradigm, consisting of training on day 1, context reexposure on day 3 and test on day 4. The duration of the reexposure session varied across experiments, lasting for 3, 5, 10, 20, or 30 min. Immediately after reexposure, the NMDA receptor antagonist MK-801 was injected intraperitoneally (0.1 mg/kg). MK-801 is a well-known amnestic agent shown to have bidirectional outcomes upon behavior when affecting reconsolidation or extinction (Lee et al. 2006; Flavell and Lee 2013; Merlo et al. 2014). The aversive response (freezing) was automatically recorded during all sessions and used as an index of fear memory.

**Figure 1.**
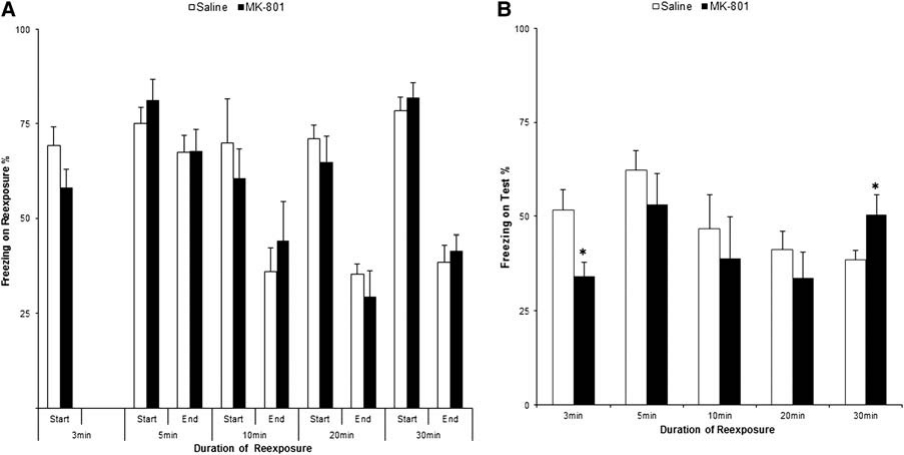
CFC memory is insensitive to MK-801 between the parameters that induce reconsolidation and extinction. Animals were subjected to Contextual Fear Conditioning and 2 d later, to a (*A*) context reexposure session of 3, 5, 10, 20, or 30-min. Immediately after, they received i.p. MK-801 or saline. Memory was assessed on the following day in a (*B*) test session. MK-801 had a significant effect when administered after 3 or 30 min, but no effect upon the intermediate conditions of 5, 10, and 20 min. There were no preexisting differences between groups during the start (first 3 min) or the end (last 3 min, or last 2 min in the 5-min condition) of the reexposure sessions in any condition. Data presented as mean + SEM. (*) *P* < 0.05 (MK-801 × Control). *n* = 14 (Sal-MK/3 min), 13 (Sal/5 min), 7 (MK/5 min), 8 (Sal-MK/10–20 min), 20 (Sal/30 min), and 22 (MK/30 min).

We found that in the short 3-min reexposure condition, while there was no difference between the experimental groups at the reexposure session itself (Fig. 1A; *F*_(1,26)_ = 2.46, *P* = 0.129; $n_{p}^{2}=0.09$; BF_10_ = 0.87), the MK-801 group showed significantly less freezing than the controls at test (Fig. 1B; *F*_(1,26)_ = 6.96, *P* = 0.014; $n_{p}^{2}=0.21$; BF_10_ = 4.00). This indicates that this short, nonreinforced context reexposure was sufficient to engage the destabilization of the previously conditioned contextual fear memory. The memory in turn, became sensitive to disruption by MK-801, resulting in impaired memory expression in the test session.

On the other hand, the administration of MK-801 after a reexposure session that lasted 10 times longer (30 min) resulted in significantly higher freezing in the test session when treated animals were compared with the control group (Fig. 1B; *F*_(1,40)_ = 4.23, *P* = 0.046; $n_{p}^{2}=0.10$; BF_10_ = 1.58). Again, there were no preexisting group differences at the beginning (*F*_(1,40)_ = 0.43, *P* = 0.517; $n_{p}^{2}=0.01$; BF_10_ = 0.36) or the end (*F*_(1,40)_ = 0.23, *P* = 0.634; $n_{p}^{2}=0.01$; BF_10_ = 0.33) of the 30-min reexposure session (Fig. 1A). These results suggest that MK-801 impaired the extinction of contextual fear memory, although the effect is rather weak (albeit statistically significant). This interpretation is consistent with the observation that context reexposure for 30 min was sufficient for a memory extinction process to take place and suppress the original CFC memory.

Interestingly, Figure 1B shows that MK-801 had no observable effect upon test freezing when administered after the intermediate context reexposures of 5 min (*F*_(1,18)_ = 0.99, *P* = 0.333; $n_{p}^{2}=0.05$; BF_10_ = 0.57), 10 min (*F*_(1,14)_ = 0.32, *P* = 0.579; $n_{p}^{2}=0.02$; BF_10_ = 0.48) and 20 min (*F*_(1,14)_ = 0.79, *P* = 0.389; $n_{p}^{2}=0.05$; BF_10_ = 0.56). Moreover, groups did not differ during the reexposure sessions in any condition, either at the beginning (5-min: *F*_(1,18)_ = 0.75, *P* = 0.398; $n_{p}^{2}=0.04$; BF_10_ = 0.53) (10-min: *F*_(1,12)_ = 0.46, *P* = 0.511; $n_{p}^{2}=0.04$; BF_10_ = 0.52) (20-min: *F*_(1,14)_ = 0.64, *P* = 0.437; $n_{p}^{2}=0.04$; BF_10_ = 0.53) or at the end (5-min: *F*_(1,18)_ = 0003, *P* = 0.957; $n_{p}^{2}=0.00$; BF_10_ = 0.41) (10-min: *F*_(1,12)_ = 0.38, *P* = 0.549; $n_{p}^{2}=0.03$; BF_10_ = 0.51) (20-min: *F*_(1,14)_ = 0.61, *P* = 0.446; $n_{p}^{2}=0.04$; BF_10_ = 0.53) of the sessions. This lack of MK-801 effect between the parameters that induced reconsolidation and extinction suggests the existence of a “null point” or “limbo” phenomenon for contextual fear memories, as shown previously in other tasks with the same drug (Flavell and Lee 2013; Merlo et al. 2014) or in CFC with a GABA-A agonist (Alfei et al. 2015).

Furthermore, by analyzing all the factors together with a two-way ANOVA, we observed a significant interaction between drug and duration of context reexposure (*F*_(4,112)_ = 2.63, *P* = 0.038; $n_{p}^{2}=0.09$; BF_10_ = 1.55), with a main effect of duration (*F*_(4,112)_ = 2.56, *P* = 0.042; $n_{p}^{2}=0.08$; BF_10_ = 1.77), but no effect of drug (*F*_(1,112)_ = 2.40, *P* = 0.124; $n_{p}^{2}=0.02$; BF_10_ = 0.38). This further strengthens the conclusion that the effect of MK-801 was dependent upon reactivation duration.

### The CFC null point is not a result of late drug administration

While the use of post-re-exposure drug administration shows the effects to be specific to reconsolidation or to the consolidation of extinction learning, it does present a potential interpretative problem. Although MK-801 had effects in both the 3- and 30-min conditions, there remained the possibility that with the intermediate exposure duration, reconsolidation was engaged, but a post-session administration of MK-801 was too late to affect the reconsolidation process (Lee and Everitt 2008). In other words, the absence of effect of MK-801 with the 10-min session might have been due to the fact that it would be too late to stop a process of memory reconsolidation that would have already happened by the time the drug became systemically available. To investigate if this could be the case, we used a 30-min prereactivation injection of MK-801, which has been demonstrated to impair reconsolidation across a number of paradigms (Lee et al. 2006; Brown et al. 2008; Flavell and Lee 2013), prior to the 10-min condition. Therefore, if reconsolidation were engaged by the 10-min context reexposure, a presession injection of MK-801 would be expected to impair reconsolidation to result in subsequent amnesia.

**Figure 2.**
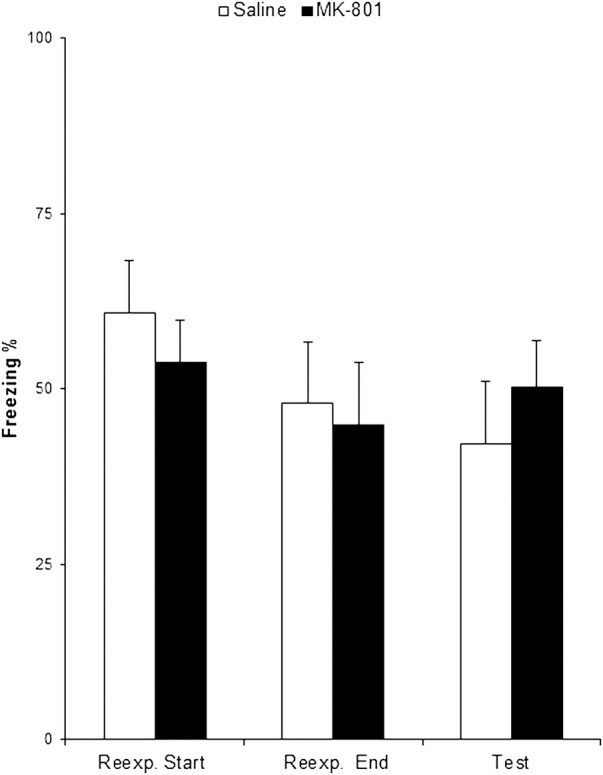
The CFC “null point” is not a result of late drug administration. Animals were subjected to contextual fear conditioning. Two days later, they received i.p. MK-801, or Saline, 30 min prior a reexposure session of 10 min. Memory was assessed on the following day in a test session. MK-801 had no significant effect. Data presented as mean + SEM. *n* = 8 per group.

Animals that received MK-801 froze at equivalent levels at test as those treated with saline (Fig. 2; *F*_(1,14)_ = 0.53, *P* = 0.478; $n_{p}^{2}=0.04$; BF_10_ = 0.51). There was no difference either at the beginning of context reexposure (*F*_(1,14)_ = 0.54, *P* = 0.473; $n_{p}^{2}=0.04$; BF_10_ = 0.51) or at the end (*F*_(1,14)_ = 0.06, *P* = 0.804; $n_{p}^{2}=0.01$; BF_10_ = 0.44). Together with the previous data (Fig. 1), it is apparent that the CFC memory was insensitive to NMDA receptor antagonism irrespective of whether MK-801 was administered prior to or immediately after a 10-min context reexposure session. Therefore, there appears to be a genuine lack of amnestic effect of MK-801 with a reexposure session of this duration.

### The CFC null point does not result from individual/subgroup differences

**Figure 3.**
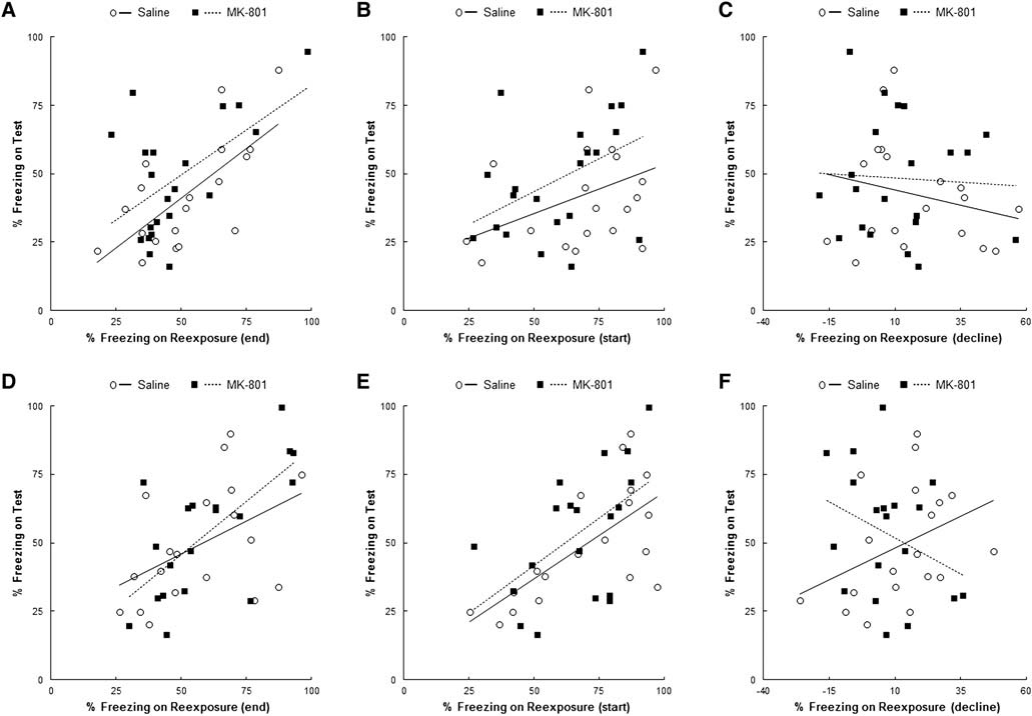
The CFC “null point” does not result from individual differences. Animals were subjected to contextual fear conditioning in big cohorts and 2 d later, to an intermediate reexposure session of 10 or 5 min. Immediately after, they received i.p. MK-801 or Saline and on the following day, memory was assessed in a test session. Freezing percentages during the test session were then correlated to (*A*) freezing at the end (last 3 min) of the 10-min reexposure session, (*B*) freezing at the start (first 3 min) of the 10-min reexposure session, (*C*) decline of freezing during the 10-min reexposure session (start–end), (*D*) freezing at the end (last 2 min) of the 5-min reexposure session, (*E*) freezing at the start (first 3 min) of the 5-min reexposure session, (*F*) decline of freezing during the 5-min reexposure session (start–end). *A*, *D,* and *E* show significant correlations between parameters for both Sal and MK-801 groups (*P* < 0.05). MK-801 did not significantly affect the slopes or the intercepts of any significant regressions. Data presented as mean + SEM. *n* = 19–21 per group.

In order to determine whether the lack of group effect of MK-801 at the intermediate 10-min condition reflects individual differences in the transition from reconsolidation to extinction, we replicated the experiment with large cohorts of rats (*n* = 19–21 per group). Our primary approach to address population effects was to focus on the correlation between freezing levels in the test and reexposure sessions. We would expect both parameters to correlate in controls, as animals with low freezing at the end of the reexposure session would be expected to freeze less in the test session as well. In contrast, if MK-801 impairs between-session extinction or reconsolidation in specific animals, we would expect an alteration of that correlation. In this case, extinction blockade would likely lead to high test freezing in animals undergoing extinction (and thus presenting lower freezing) during the reexposure session, while animals undergoing reconsolidation (with presumably high freezing during context reexposure) would be expected to freeze less at the test. Again, MK-801 did not have any effect when analyzing the population as a whole (*F*_(1,38)_ = 0.85, *P* = 0.362; $n_{p}^{2}=0.02$; BF_10_ = 0.43) (Supplemental Fig. S2a). When we plot the freezing levels of animals at the end of context reexposure (as an index of within-session extinction) and test (as an index of between-session extinction), we indeed observe a positive correlation between sessions for the control animals (Fig. 3A; *r* = 0.683*, P* = 0.001, BF_10_ = 35.14). This correlation was not disrupted by the administration of MK-801 (*r* = 0.534*, P* = 0.013, BF_10_ = 4.99), and the slopes (*F*_(1,36)_ = 0.09, *P* = 0.768) and intercepts (*F*_(1,37)_ = 2.51, *P* = 0.121) of the two linear regressions were not statistically different.

Additional analyses compared freezing at test to freezing at the start of context reexposure (Fig. 3B), showing a positive, but nonsignificant correlation for both control (*r* = 0.381*, P* = 0.107, BF_10_ = 0.95) and MK-801-treated (*r* = 0.444*, P* = 0.444, BF_10_ = 1.83) groups. A comparison of the slopes of these nonsignificant correlations revealed no difference in their slopes (*F*_(1,36)_ = 0.16, *P* = 0.692) and intercepts (*F*_(1,37)_ = 2.61, *P* = 0.115). Given that freezing at the start of context reexposure is variable across rats, it is possible that an index of the decline in freezing over the course of the session is a more reliable measure of within-session extinction, and therefore we correlated such an index with test freezing (Fig. 3C). Surprisingly, there was no significant correlation, either for animals that received saline (*r* = −0.230*, P* = 0.344, BF_10_ = 0.43) or MK-801(*r* = −0.044*, P* = 0.848, BF_10_ = 0.27). For completeness, we compared the slopes and intercepts of these nonsignificant linear regressions, which revealed no difference in their slopes (*F*_(1,36)_ = 0.25, *P* = 0.616) and intercepts (*F*_(1,37)_ = 0.61, *P* = 0.439). Finally we correlated test freezing with performance in the elevated plus maze task, performed 1 wk before fear conditioning, in order to test whether baseline anxiety levels affected the impact of MK-801 (Supplemental Fig. S1). Again, no correlation was observed in either of the groups (Sal: *r* = 0.206*, P* = 0.398, BF_10_ = 0.40; MK-801: *r* = 0.057*, P* = 0.806, BF_10_ = 0.28) and slopes and intercepts did not differ (*F*_(1,36)_ = 0.17, *P* = 0.682 and *F*_(1,37)_ = 0.81, *P* = 0.373, respectively).

In order to confirm the findings of our analyses for the 10-min condition, we replicated them on a large cohort tested with the 5-min reexposure that also appears to fall within the null point (but without the baseline elevated plus maze). Once more, MK-801 did not have any effect when analyzing the population as a whole (*F*_(1,36)_ = 0.35, *P* = 0.558; $n_{p}^{2}=0.01$; BF_10_ = 0.36) (Supplemental Fig. S3a). Moreover, we observed a pattern of results similar to that observed with the 10-min analyses (Fig. 3D–F), indicating that there were no subpopulations of reconsolidating and extinguishing rats. Freezing at the end of context reexposure and test correlated positively for the control (Fig. 3D: *r* = 0.457*, P* = 0.049, BF_10_ = 1.72) and MK-801 animals (*r* = 0.683*, P* = 0.001, BF_10_ = 35.07), with the two linear regressions not differing in comparison of their slopes (*F*_(1,34)_ = 0.92, *P* = 0.344) and intercepts (*F*_(1,35)_ = 0.17, *P* = 0.681). Positive correlations were also observed between freezing at the start of reexposure and in the test session, and were more robust than those seen with 10-min condition (Sal: *r* = 0.682*, P* = 0.001, BF_10_ = 34.45; MK-801: *r* = 0.530*, P* = 0.020, BF_10_ = 3.58). Nevertheless, there was no significant difference in slope (*F*_(1,34)_ = 0.04, *P* = 0.850) or intercept (*F*_(1,35)_ = 1.09, *P* = 0.302) of the two linear regressions. Finally, there was again no significant correlation between freezing decline across the brief reexposure session and freezing at test in either of the groups (Sal: *r* = 0.379*, P* = 0.110, BF_10_ = 0.93; MK-801: *r* = −0.326*, P* = 0.173, BF_10_ = 0.67). While the statistical comparison between slopes revealed a significant difference (*F*_(1,34)_ = 4.70, *P* = 0.037; the magnitude of the difference does not allow the comparison of intercepts), this is likely a chance finding, as both correlations are weak and nonsignificant, as observed in the 10-min condition.

As an alternative analytical approach, we stratified the large cohorts of rats, for both the 10- and 5-min conditions, into subgroups, based upon baseline anxiety (high versus low), freezing at the start of the reexposure session (high versus low), freezing at the end of reexposure (high versus low), freezing decline across the reexposure session (small versus large) and freezing during the test itself (high versus low). Thereafter, CFC memory and the effect of MK-801 on the test session were analyzed across the different subpopulations of animals. None of these subpopulation analyses suggested the existence of divergent effects of MK-801 in different individuals (Supplemental Figs S2, S3).

Finally, we compared the variability in test freezing between the MK-801 and saline control groups for both the 10- and 5-min larger cohort experiments. Levene's test revealed that there was no difference between the groups’ variances, either in the 10-min (*F*_(1,38)_ = 0.34, *P* = 0.562) or in the 5-min condition (*F*_(1,36)_ = 0.26, *P* = 0.610). The fact that the test variances between the saline and MK-801 groups are similar offers further evidence against the idea that MK-801 could be affecting reconsolidation in some animals and extinction in others during the null-point.

**Figure 4.**
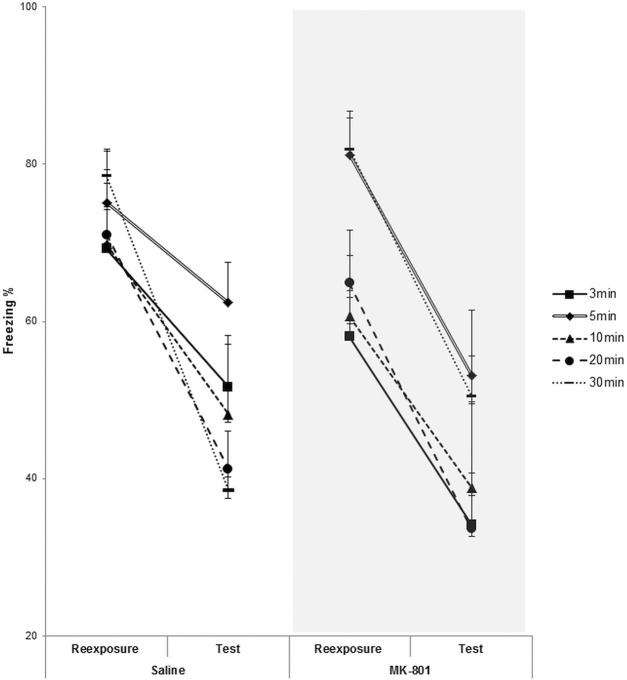
Consistent reductions in freezing from reexposure to test. Analysis of the freezing at the start of context reexposure (first 3-min) and at test from the experiment in Figure 1. There were consistent reductions in freezing in all conditions (except for Sal 5-min). Data is presented as mean + SEM. *n* = 14 (Sal-MK/3 min), 13 (Sal/5 min), 7 (MK/5 min), 8 (Sal-MK/10–20 min), 20 (Sal/30 min), and 22 (MK/30 min).

While there was no evidence for subpopulation differences in susceptibility to reconsolidation or extinction at intermediate reexposure durations, the analyses revealed an interesting pattern of consistent reductions in freezing from context reexposure to test, regardless of the duration of the reexposure. When comparing freezing between the start of reexposure and the test session in control rats from the first experiment (Figs. 1, 4), a repeated-measures ANOVA revealed a main effect of session (*F*_(1,57)_ = 87.24, *P* < 0.001, $n_{p}^{2}=0.61$; BF_10_ = 3.97), no effect of reexposure duration (*F*_(4,57)_ = 1.37, *P* = 0.256, $n_{p}^{2}=0.09$; BF_10_ = 0.17) and a significant duration × session interaction (*F*_(4,57)_ = 4.94, *P* = 0.002, $n_{p}^{2}=0.26$; BF_10_ = 6.51). Analyses of simple main effects confirmed a reduction in freezing with the longer 10-, 20-, and 30-min conditions (*P* < 0.05, $n_{p}^{2}>0.60$; BF_10_ > 2.00). While there was only a marginal effect of reduced freezing with the 5-min reexposure (*P* = 0.080, $n_{p}^{2}=0.23$; BF_10_ = 1.89), there was a significant reduction after the 3-min condition (*P* < 0.001, $n_{p}^{2}=0.75$; BF_10_ = 368.59), indicating that context reexposure without reinforcement can result in some degree of decrease in freezing from reexposure to test even with short durations. By performing the same comparisons in the MK-801 animals, we observe a main effect of session (*F*_(1,54)_ = 82.06, *P* < 0.010, $n_{p}^{2}=0.59$; BF_10_ = 1.31), an effect of reactivation duration (*F*_(4,54)_ = 4.15, *P* = 0.005, $n_{p}^{2}=0.24$; BF_10_ = 4.83) and no significant duration × session interaction (*F*_(4,54)_ = 0.50, *P* = 0.738, $n_{p}^{2}=0.01$; BF_10_ = 2.57). Analyses of simple main effects confirmed a reduction in freezing from reactivation to test with all reactivation durations (*P* < 0.010, $n_{p}^{2}>0.55$; BF_10_ > 5.00). It is notable that even with the 30-min condition, in which MK-801 animals froze significantly more than the controls (Fig. 1B), a decrease in memory expression from reactivation to test was still observed. Moreover, the freezing behavior in nonreactivation control groups (Sal = 74 ± 5, *n* = 19) (MK-801 = 71 ± 5, *n* = 17) confirmed that reactivation of any duration resulted in decreased freezing at test, in spite of MK-801 treatment.

## Discussion

In this study, we have demonstrated that MK-801 impaired contextual fear memory reconsolidation with a short reexposure duration, and disrupted extinction with a long reexposure duration, as shown previously with other drugs in contextual fear conditioning (Suzuki et al. 2004; Bustos et al. 2009; Alfei et al. 2015) and with the same drug in other paradigms (Lee et al. 2006; Merlo et al. 2014). At intermediate durations of context reexposure, MK-801 had no observable effect on the expression of the contextual fear memory. This lack of effect was not due to the timing of MK-801 administration, as it was replicated with presession injection of the drug. Moreover, there was no evidence for subpopulations of animals responding differently to MK-801 at the intermediate reexposure duration. These results suggest that there is a period during the transition from reconsolidation to extinction where memory is indeed not sensitive to disruption.

The null point between reconsolidation and extinction has previously been demonstrated for appetitive Pavlovian memories (Flavell and Lee 2013) and cued fear memories (Merlo et al. 2014), as well as for the contextual fear memories studied here (Alfei et al. 2015). In each of these studies, one intermediate parameter of memory reactivation was found, in which amnestic treatment did not either impair or enhance subsequent memory expression at test. While two of the previous studies used the same dose of MK-801 as used here (Flavell and Lee 2013; Merlo et al. 2014), the third used systemic injections of midazolam (Alfei et al. 2015). Therefore, the existence of the null point is not unique to the use of MK-801 or to NMDA receptor antagonists.

**Figure 5.**
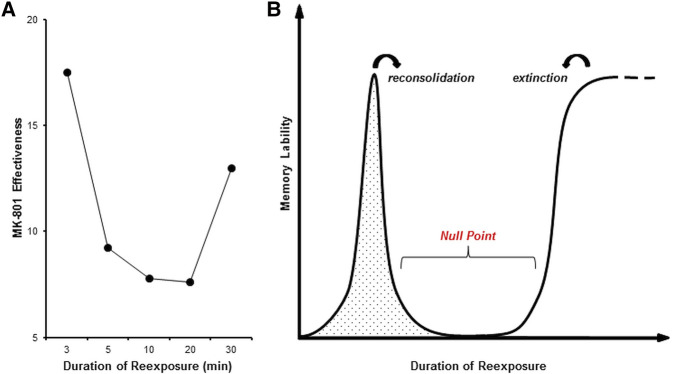
Three phase transition from reconsolidation to extinction. (*A*) Effectiveness of amnesic treatment (absolute difference in freezing between MK-801 and saline groups during test) across different conditions reveals a (*B*) three-phase model for the transition of reconsolidation to extinction of associative memories.

In the current study, there was evidence for an extended null point period between the reexposure durations that induce reconsolidation and extinction. Context reexposures of 5, 10, and 20 min each revealed a lack of effect of MK-801. This extended duration in itself suggests that the null point cannot be explained simply by variability in the point of transition between reconsolidation and extinction across different animals, as one would expect at least some trend toward reconsolidation impairment at the 5-min end and extinction impairment at the 20-min end. Moreover, we predicted that the existence of subgroups showing impaired reconsolidation or extinction would manifest as (a) a reduction in the correlation between freezing levels in the reexposure and test sessions in treated animals, (b) an effect of MK-801 when analyzing subpopulations determined by one or more factors (e.g., levels of freezing at context reexposure or test, or extent of within-session extinction during reexposure), or (c) increased variability in the MK-801-treated rats compared with saline-treated controls. None of these predictions were supported by our data. Therefore, we conclude that the null point represents a period at which MK-801 impairs neither reconsolidation nor extinction (Fig. 5), in accordance to the three-phase transition model outlined by Merlo et al. (2014).

Contextual fear memory reconsolidation and extinction have both been demonstrated to be critically dependent upon NMDA receptor activity (Suzuki et al. 2004; Lee and Hynds 2013; Lee and Flavell 2014). The bidirectional effect of the same amnestic treatment, dependent upon the parameters of memory reactivation, indicates that the dissociable effects are mediated by impairments in different mnemonic processes (Lee et al. 2006; de la Fuente et al. 2011). This has led to the suggestion that there is a trace dominance effect, with the trace that is dominantly activated by memory retrieval being the one impaired by amnestic treatment, such as NMDA receptor antagonism or protein synthesis inhibition (Eisenberg et al. 2003). Mechanisms for such trace dominance have been postulated by computational models, in which different degrees of similarity between training and reexposure lead to reconsolidation or extinction-like phenomena (Osan et al. 2011; Gershman et al. 2017). However, these models do not predict a null point in which neither reconsolidation nor extinction is dominantly activated, leading to the lack of effect of MK-801. Our data, on the contrary, indicate that under conditions of no dominant trace activation, there is no disruptive effect of MK-801 on either reconsolidation or extinction in individual animals. Therefore, the start of NMDA receptor-dependent extinction per se does not seem to be the factor preventing memory reconsolidation. Instead, it appears that there could be independent mechanisms that suppress the engagement of reconsolidation, but are not by themselves sufficient to engage extinction. This may well be mediated at the cellular level (de la Fuente et al. 2011; Merlo et al. 2014), although we cannot rule out the possibility that the complex interplay between reconsolidation and extinction is regulated at the systems level, especially given that reconsolidation and extinction have only partially overlapping neural substrates (Bahar et al. 2004).

The current results also reveal a dissociation between the definitions of extinction as new learning vulnerable to amnestic treatment (e.g., MK-801) and as a long-term reduction of memory expression after reexposure (Pavlov 1927). It was notable that all reexposure durations resulted in a decline in contextual fear memory expression in the test session (Fig. 4). This was not due simply to the passage of time, as nonreexposed controls froze at higher levels than those undergoing reexposure. Therefore, while context reexposure led to behaviorally defined extinction for all durations, this extinction was apparently NMDA receptor-dependent only for the 30-min condition. Moreover, the reconsolidation impairment with the brief reexposure duration was observed in spite of a significant between-session decline in freezing, as has been previously documented in the literature (Charlier and Tirelli 2011; Brabant et al. 2013; Heath et al. 2015). Importantly, we observed a similar pattern of freezing reduction even in the animals that received MK-801 after context reexposure, no matter how long the session lasted for (Fig. 4). This extends even to the 30-min duration, indicating the presence of an NMDA receptor-independent process that weakens memory expression in these conditions. Indeed, the data from Merlo et al. (2014) show the same pattern of between-session memory decline in cued fear that was unaffected by MK-801 at their intermediate null point parameter. The fact that some degree of behaviorally defined extinction occurs in the absence of NMDA activity raises the question of what causes this freezing decline. Although it could be related to non-NMDA receptor-dependent extinction learning, which has been described in some conditions (Santini et al. 2001; Langton and Richardson 2008; Kim and Richardson 2010), or to delayed consolidation of extinction (i.e., beyond systemic availability of MK-801), it might also imply that behavioral extinction, at least in some cases, might involve not only learning of a new association, but also weakening of the original one (Barad 2006; Riebe et al. 2012; Almeida-Corrêa and Amaral 2014) through a process that might be less dependent on NMDA receptors than new learning.

Regardless of the uncertainty about the potential multiple mechanisms of weakening memory expression with extinction training, these observations and our wider results raise an important point about the transition between reconsolidation, the null point, and extinction. We could detect no reliable basis, other than systematically varying duration of context reexposure, upon which to predict whether a given duration will engage reconsolidation, NMDA receptor-dependent extinction or will fall into the null point. Certainly, there is no obvious pattern or threshold of memory decline that can distinguish between the parameters leading to reconsolidation and extinction. Previous studies of contextual fear memory showed that the parameters of the three-phase transition were partly dependent upon the timing of shock delivery during conditioning (Alfei et al. 2015), and that the parameters of reconsolidation depend upon memory age and strength (Suzuki et al. 2004). Although this was not tested directly, it is reasonable to predict that if older and stronger memories require more extended context reexposure to induce reconsolidation (Suzuki et al. 2004), the parameters of the null point and extinction will be similarly shifted to longer durations.

Previous studies have suggested a critical role for prediction error in triggering reconsolidation across a number of paradigms (Reichelt and Lee 2012; Díaz-Mataix et al. 2013; Reichelt et al. 2013; Sevenster et al. 2013; Alfei et al. 2015), a finding that has also been incorporated by computational models (Osan et al. 2011; Gershman et al. 2017). However, with increasing nonreinforced stimulus reexposure, it is unlikely that there is a sufficient qualitative or quantitative change in the prediction error signal to explain the transition to the null point and beyond to the NMDA receptor-dependent extinction phase. Moreover, it is not obvious how the instantiation of prediction error-mediated learning, for example, within the Rescorla–Wagner rule (Rescorla and Wagner 1972), is consistent with the new learning that is characteristic of extinction, rather than prediction error-mediated memory weakening (Exton-McGuinness et al. 2015). Indeed, it is possible, and perhaps likely, that there are independent mechanisms controlling destabilization and NMDA-receptor dependent extinction (Exton-McGuinness et al. 2015). While a sharp transition between reconsolidation and extinction might suggest a direct interaction between the two, the three-phase transition with an intermediate null point may reflect the independent control of reconsolidation and extinction. Indeed, it could also explain occurrences when there appears to be no competition between reconsolidation and extinction (Duvarci et al. 2006). In such cases, destabilization/reconsolidation might be triggered regardless of the extent of stimulus reexposure, and may even overlap with the engagement of extinction.

In conclusion, our results demonstrate that during the retrieval of contextual fear memories, there is a genuine “null point” between the parameters that induce reconsolidation and extinction, at which the memory is not sensitive to disruption by MK-801. Nevertheless, context reexposure can still lead to NMDA receptor-independent decreases in freezing during this null point. These findings reinforce and expand the hypothesis of a three-phase transition between reconsolidation and extinction of associative memories, bringing new insights on the different ways a mnemonic trace might be affected by memory retrieval.

## Materials and Methods

### Subjects

Subjects were 228 experimentally naïve adult male Lister Hooded rats (200–350 g at the start of the experiment) from Charles River (UK). Animals were housed in groups of four per cage, under a 12 h light–dark cycle (lights on at 07:00) and a 21°C temperature, with water and food provided ad libitum apart from during the behavioral sessions. Cages contained aspen chip bedding, and environmental enrichment was available in the form of a Plexiglas tunnel. Experiments took place in a behavioral laboratory between 10:00 and 14:00. At the end of the experiment, animals were humanely killed via a rising concentration of CO_2_; death was confirmed by cessation of heartbeat. All procedures were approved by a local ethical review committee and conducted in accordance to the United Kingdom Animals (Scientific Procedures) Act 1986, Amendment Regulations 2012 (PPL 70/7662).

### Behavioral apparatus

The conditioning chambers (MedAssociates) consisted of two identical illuminated boxes (25 cm × 32 cm × 25.5 cm), placed within sound-attenuating chambers. The box walls were constructed of steel, except by the ceiling and front wall, which were made of Perspex. The grid floor consisted of 19 stainless steel rods (4.8 mm diameter; 1.6 mm center to center), connected to a shock generator and scrambler (MedAssociates). Infrared video cameras were mounted on the ceiling of the chambers (Viewpoint Life Sciences) and used to record behavior.

### Contextual fear conditioning

During the training session, rats were placed individually in the conditioning chambers. After 3 min of free exploration, animals received 2 footshocks (0.7 mA, 1.5 sec) separated by a 30-sec interval, and after 1 min, were placed back into their home cages. Two days later, animals were reexposed to the same context for 3, 5, 10, 20, or 30 min. One day later, animals were exposed one more time to the context for 3 min, in order to assess memory expression (test session). No footshock was applied at either reexposure or test sessions. The aversive response (freezing) was automatically quantified during all sessions with a videotracking software (Viewpoint Life Sciences), and used as a memory index (Lee and Hynds 2013; Song et al. 2016).

### Elevated plus maze

A standard maze composed of two open arms and two closed arms from MedAssociates was used. The rats were placed individually in the center of the maze, facing an open arm, and allowed 10 min of free exploration. Time spent in the open arms was scored manually by a researcher based outside the experimental room, and used as an index for baseline anxiety. Animals were considered to be in the arm when all four paws were placed within (Hu et al. 2014).

### Drugs

MK-801 (Sigma-Aldrich) was diluted in sterile saline (0.1 mg/mL) and injected intraperitoneally (1 mL/kg) immediately after the reexposure session, or 30 min previously when specified (Lee et al. 2006; Song et al. 2016). Injections of MK-801 or vehicle were randomly distributed between animals.

### Statistics

Data were analyzed in JASP (JASP Team 2017). Between-group comparisons were performed with one-way or two-way ANOVA, where needed. For within-group comparisons, repeated-measures ANOVA was applied. Levene's test was used for comparison of variability between groups. For slope and interception comparisons of linear regressions, data were analyzed in Prism (GraphPad Software 2017). Significance was always set at *P* < 0.05 and data are presented as mean + SEM. Animals freezing more than 95% during the context reexposure sessions were excluded from analysis (five animals). The rationale for this was that asymptotic learning appears to result in a resistance to memory destabilization (Rodriguez-Ortiz et al. 2005, 2008; Lee 2010), and so rats that froze at near-maximal levels during context reexposure would be unlikely to undergo reconsolidation regardless of reexposure duration. Similarly, animals that do not learn at all would not be suited to detect reconsolidation or extinction impairments, and so a criterion of >5% freezing was also imposed, although this did not result in the exclusion of any animals. $n_{p}^{2}$ was used as an estimate of effect size and BF_10_ is also reported as the outcome of Bayesian analyses for the estimation of posterior probability (Jarosz and Wiley 2014).

## Supplementary Material

Supplemental Material
