# Supplementary material for: On the transition from reconsolidation to extinction of contextual fear memories
Source: Learn Mem. 2017 Sep;24(9):392–9. doi: 10.1101/lm.045724.117 (PMC5580521; doi:10.1101/lm.045724.117)
Supplement: Supplemental Material [file supp_24_9_392__index.html]

Supplemental Material 

# On the transition from reconsolidation to extinction of contextual fear memories

## Supplemental Material

- Suplemental\_Figure\_1.pdf
- Suplemental\_Figure\_2.pdf
- Suplemental\_Figure\_3.pdf
