# Supplementary material for: On the transition from reconsolidation to extinction of contextual fear memories
Source: Learn Mem. 2017 Sep;24(9):392–9. doi: 10.1101/lm.045724.117 (PMC5580521; doi:10.1101/lm.045724.117)
Supplement: Supplemental Material [file supp_24.9.392_Suplemental_Figure_1.pdf]

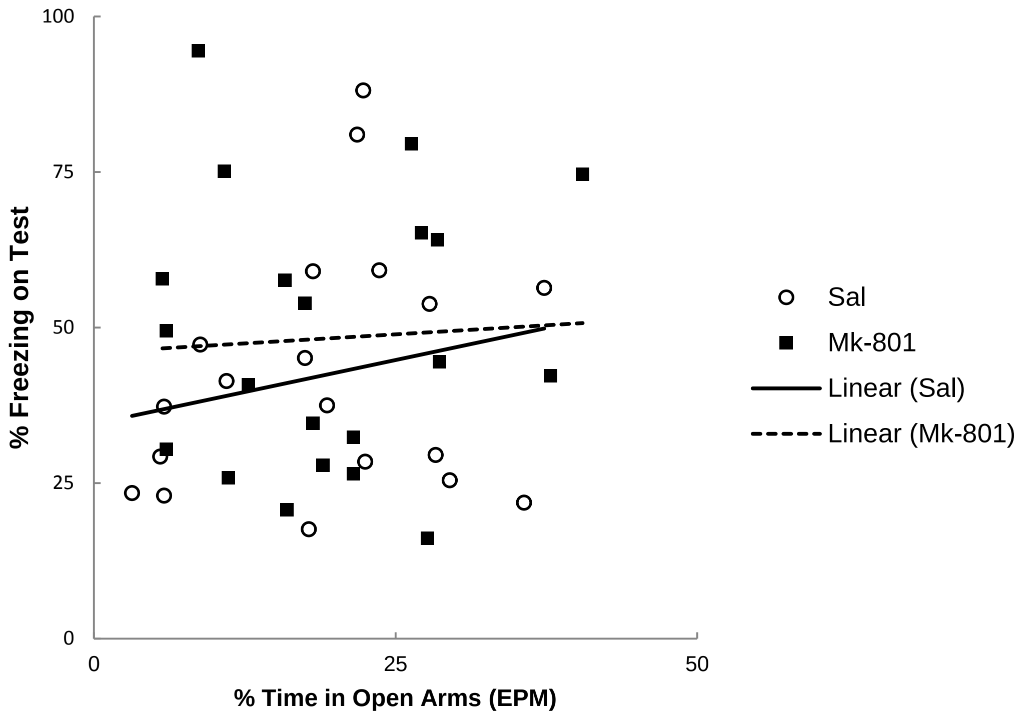

Fig. S1. Baseline anxiety does correlate with freezing during CFC test. Animals were placed individually in an Elevated plus maze (EPM) and allowed 10 min of free exploration. Time spent in the open arms was used as index for baseline anxiety and correlated against the percent time freezing during the contextual fear memory test. No correlation was observed in either of the groups ($p > 0.390$). Additional correlations are depicted in Figure 3. $n = 19\text{-}21$ per group.
