# Supplementary material for: On the transition from reconsolidation to extinction of contextual fear memories
Source: Learn Mem. 2017 Sep;24(9):392–9. doi: 10.1101/lm.045724.117 (PMC5580521; doi:10.1101/lm.045724.117)
Supplement: Supplemental Material [file supp_24.9.392_Suplemental_Figure_2.pdf]

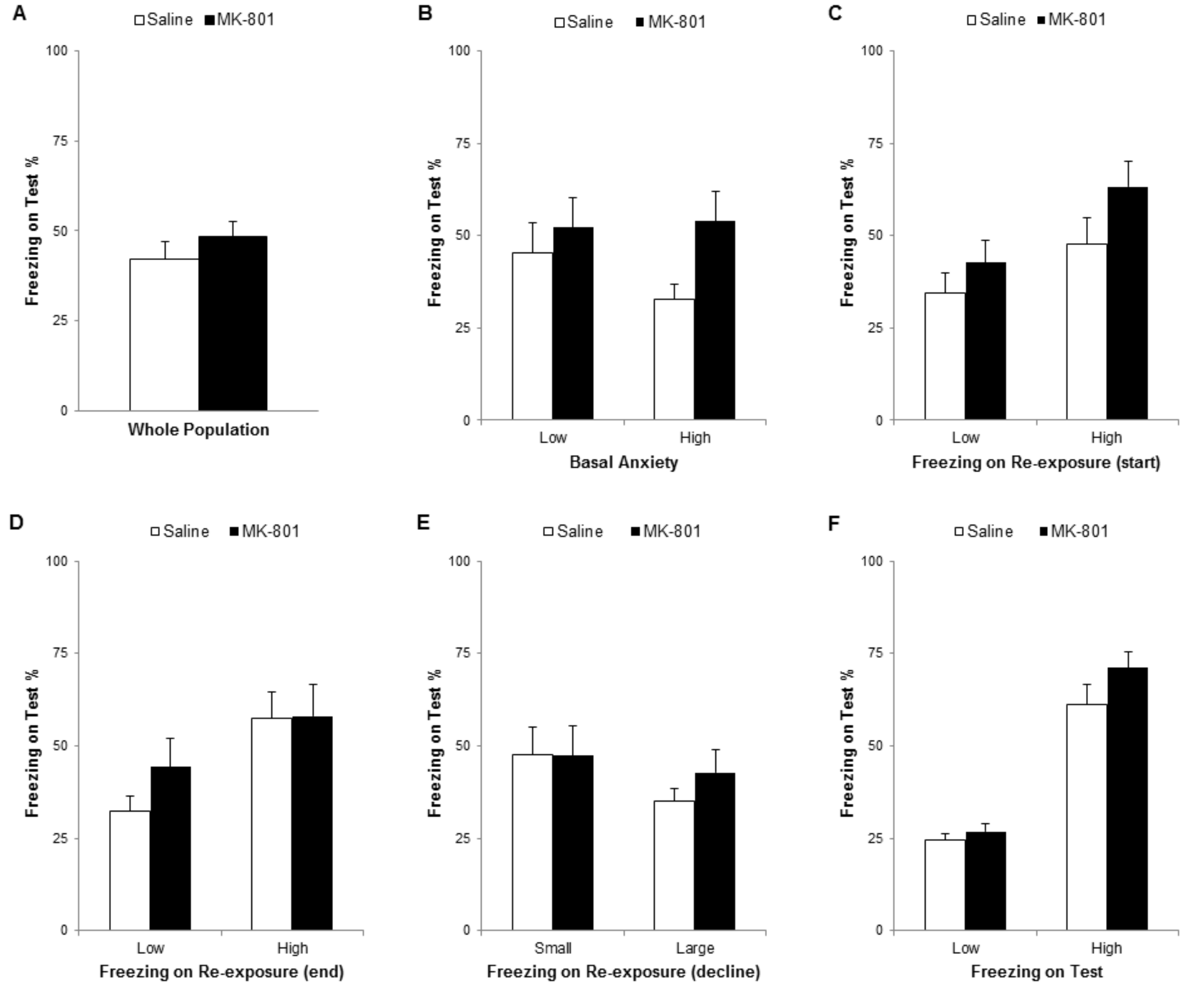

Figure S2. Subgroup analysis of animals undergoing 10-min context re-exposure. Animals were subjected to contextual fear conditioning in big cohorts and, two days later, to an intermediate re-exposure session of 10-min. Immediately after, they received i.p MK-801 or saline, and on the following day memory was assessed in a test session. Animals were then allocated to two different groups, according to the following parameters: basal anxiety, freezing at the start (first 3min) and at the end (last 3min) of the context re-exposure, freezing decline across re-exposure session (start – end) and freezing at test. For each parameter, values from subjects of each group (saline & MK-801) were ordered from smallest to largest. The top 8 animals were allocated to one group (e.g. low freezers) and the bottom 8 to the opposite (e.g. high freezers). Thereafter, the differential effect of MK-801 was analysed on these subpopulations. **(A)** MK-801 did not have any effect when analysing the population as a whole ($F_{1,38}$ = 0.85, p = 0.362; η^2_p = 0.02; BF_{10} = 0.43). Moreover, two-way ANOVA analysis revealed that the MK-801 effect did not depend upon **(B)** the baseline anxiety of animals (drug: $F_{1,28}$ = 3.76, p = 0.062, η^2_p = 0.12; BF_{10} = 1.39; subpopulation: $F_{1,28}$ = 0.56, p = 0.463, η^2_p = 0.02; BF_{10} = 0.41; drug x subpopulation: $F_{1,28}$ = 0.94, p = 0.340, η^2_p = 0.03; BF_{10} = 0.33). **(C)** The level of freezing during the start of the re-exposure session did not seem to be an important factor either (drug: $F_{1,28}$ = 3.33, p = 0.079, η^2_p = 0.11; BF_{10} = 0.98; subpopulation: $F_{1,28}$ = 6.72, p = 0.015, η^2_p = 0.19; BF_{10} = 3.39; drug x subpopulation: $F_{1,28}$ = 0.31, p = 0.582, η^2_p = 0.01; BF_{10} = 2.25). **(D)** There was also no effect of MK-801 regardless of the freezing level of individuals at the end of the re-exposure session (drug: $F_{1,28}$ = 0.81, p = 0.377, η^2_p = 0.03; BF_{10} = 0.43; subpopulation: $F_{1,28}$ = 7.73, p = 0.010, η^2_p = 0.22; BF_{10} = 5.61; drug x subpopulation: $F_{1,28}$ = 0.68, p = 0.416, η^2_p = 0.02; BF_{10} = 1.39). These analyses did, however, confirm that rats that froze more at the start or end of context re-

exposure also froze more at the subsequent test. **(E)** Furthermore, MK-801 did not have any effect no matter whether animals presented a small or a large decline of freezing across the re-exposure session (drug: $F_{1,28} = 0.34$, $p = 0.567$, $\eta^2_p = 0.01$; $BF_{10} = 0.38$; subpopulation: $F_{1,28} = 1.69$, $p = 0.204$, $\eta^2_p = 0.06$; $BF_{10} = 0.66$; drug x subpopulation: $F_{1,28} = 0.38$, $p = 0.542$, $\eta^2_p = 0.01$; $BF_{10} = 0.12$). **(F)** Finally, considering individual differences during the test itself, we also observed that MK-801 exerted no effect upon memory either on high- or low-freezing animals (drug: $F_{1,28} = 2.66$, $p = 0.114$, $\eta^2_p = 0.09$; $BF_{10} = 0.41$; subpopulation: $F_{1,28} = 117.42$, $p < 0.001$, $\eta^2_p = 0.81$; $BF_{10} = 3.79$; drug x subpopulation: $F_{1,28} = 1.12$, $p = 0.299$, $\eta^2_p = 0.04$; $BF_{10} = 1.92$). There was no evidence for MK-801 either impairing reconsolidation to reduce freezing below the level of equivalent subpopulation control animals, or disrupting extinction to increase freezing above the level of equivalent controls. Therefore, none of these analyses support the hypothesis that there are inter-individual differences in the response to MK-801, and instead are more consistent with the existence of a null point at the individual level. Data are presented as mean + SEM. n = 19-21 per group / 8 per subgroup.
