# Supplementary material for: On the transition from reconsolidation to extinction of contextual fear memories
Source: Learn Mem. 2017 Sep;24(9):392–9. doi: 10.1101/lm.045724.117 (PMC5580521; doi:10.1101/lm.045724.117)
Supplement: Supplemental Material [file supp_24.9.392_Suplemental_Figure_3.pdf]

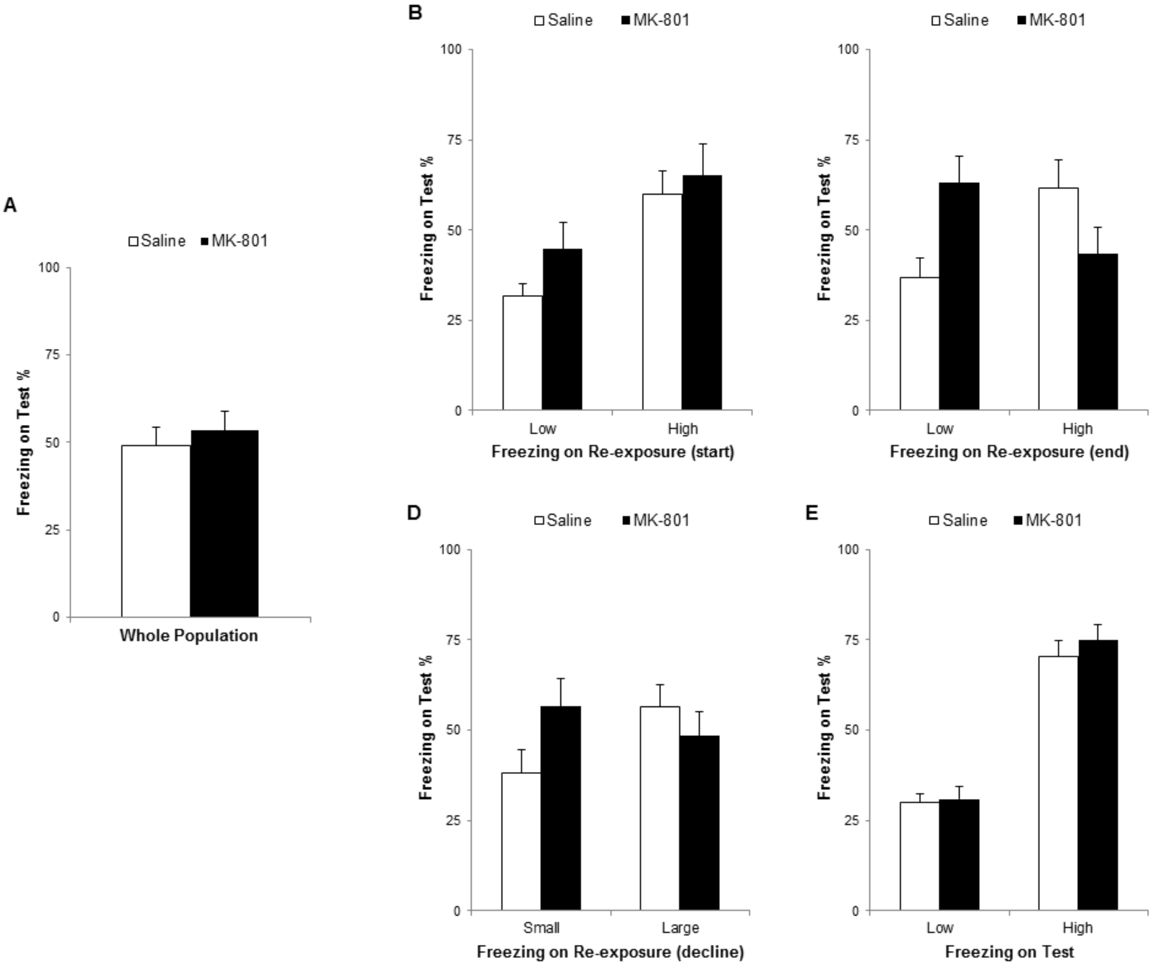

Figure S3. Subgroup analysis of animals undergoing 5-min context re-exposure. Animals were subjected to contextual fear conditioning in big cohorts and, two days later, to an intermediate reactivation session of 5-min. Immediately after, they received i.p MK-801 or saline, and on the following day memory was assessed in a test session. Animals were then allocated to two different groups, according to the following parameters: freezing at the start (first 3min) and at the end (last 2min) of the context re-exposure , freezing decline across context re-exposure (start – end) and freezing at test. For each parameter, values from subjects of each group (saline & MK-801) were ordered from smallest to largest. The top 8 animals were allocated to one group (e.g. low freezers) and the bottom 8 to the opposite (e.g. high freezers). Thereafter, the differential effect of MK-801 was analysed on these subpopulations. **(A)** MK-801 did not have any effect when analysing the population as a whole ($F_{1,36}$ = 0.35, p = 0.558; η^2_p = 0.01; BF_{10} = 0.36). Moreover, two-way ANOVA analysis revealed that the MK-801 effect did not depend upon **(B)** the level of freezing during the start of the re-exposure session (drug: $F_{1,28}$ = 1.75, p = 0.197, η^2_p = 0.06; BF_{10} = 0.55; subpopulation: $F_{1,28}$ = 12.34, p = 0.002, η^2_p = 0.31; BF_{10} = 23.03; drug x subpopulation: $F_{1,28}$ = 0.31, p = 0.582, η^2_p = 0.01; BF_{10} = 6.87). **(C)** There was a significant interaction between drug and subgroup based on the level of freezing at the end of re-exposure (drug: $F_{1,28}$ = 0.32, p = 0.577, η^2_p = 0.01; BF_{10} = 0.37; subpopulation: $F_{1,28}$ = 0.14, p = 0.714, η^2_p = 0.01; BF_{10} = 0.35; drug x subpopulation: $F_{1,28}$ = 9.85, p = 0.004, η^2_p = 0.26; BF_{10} = 1.45). However, this interaction is likely not meaningful as it is also seen at the start ($F_{1,28}$ = 13.90, $p < 0.001$, η^2_p = 0.33; BF_{10} = 14.82) and at the end ($F_{1,28}$ = 21.97, $p < 0.001$, η^2_p = 0.44; BF_{10} = 172.42) of re-exposure , probably reflecting a pre-existing difference rather than an MK-801-induced difference. **(D)** Furthermore, MK-801 did not have any effect no matter whether

animals presented a small or a large decline of freezing across the re-exposure session (drug: $F_{1,28}$ = 0.57, p = 0.455, η^2_p = 0.02; BF_{10} = 0.41; subpopulation: $F_{1,28}$ = 0.55, p = 0.466, η^2_p = 0.02; BF_{10} = 0.41; drug x subpopulation: $F_{1,28}$ = 3.76, p = 0.063, η^2_p = 0.12; BF_{10} = 0.25). **(E)** Finally, considering individual differences during the test itself, we also observed that MK-801 exerted no effect upon memory either on high- or low-freezing animals (drug: $F_{1,28}$ = 0.49, p = 0.490, η^2_p = 0.02; BF_{10} = 0.35; subpopulation: $F_{1,28}$ = 119.95, p < 0.001, η^2_p = 0.81; BF_{10} = 1.51; drug x subpopulation: $F_{1,28}$ = 0.25, p = 0.618, η^2_p = 0.01; BF_{10} = 2.69). There was no reliable evidence for MK-801 either impairing reconsolidation to reduce freezing below the level of equivalent subpopulation control animals, or disrupting extinction to increase freezing above the level of equivalent controls. Therefore, these analyses do not support the hypothesis that there are inter-individual differences in the response to MK-801, and instead are more consistent with the existence of a null point at the individual level. Data are presented as mean + SEM. n = 19 per group / 8 per subgroup.
